# Origins and Early Evolution of the tRNA Molecule

**DOI:** 10.3390/life5041687

**Published:** 2015-12-03

**Authors:** Koji Tamura

**Affiliations:** 1 Department of Biological Science and Technology, Tokyo University of Science, 6-3-1 Niijuku, Katsushika-ku, Tokyo 125-8585, Japan; koji@rs.tus.ac.jp; Tel.: +81-3-5876-1472; 2 Research Institute for Science and Technology, Tokyo University of Science, 2641 Yamazaki, Noda, Chiba 278-8510, Japan

**Keywords:** tRNA, minihelix, origin, evolution, genetic code

## Abstract

Modern transfer RNAs (tRNAs) are composed of ~76 nucleotides and play an important role as “adaptor” molecules that mediate the translation of information from messenger RNAs (mRNAs). Many studies suggest that the contemporary full-length tRNA was formed by the ligation of half-sized hairpin-like RNAs. A minihelix (a coaxial stack of the acceptor stem on the T-stem of tRNA) can function both in aminoacylation by aminoacyl tRNA synthetases and in peptide bond formation on the ribosome, indicating that it may be a vestige of the ancestral tRNA. The universal CCA-3′ terminus of tRNA is also a typical characteristic of the molecule. “Why CCA?” is the fundamental unanswered question, but several findings give a comprehensive picture of its origin. Here, the origins and early evolution of tRNA are discussed in terms of various perspectives, including nucleotide ligation, chiral selectivity of amino acids, genetic code evolution, and the organization of the ribosomal peptidyl transferase center (PTC). The proto-tRNA molecules may have evolved not only as adaptors but also as contributors to the composition of the ribosome.

## 1. Origins of tRNA

**Figure 1:**
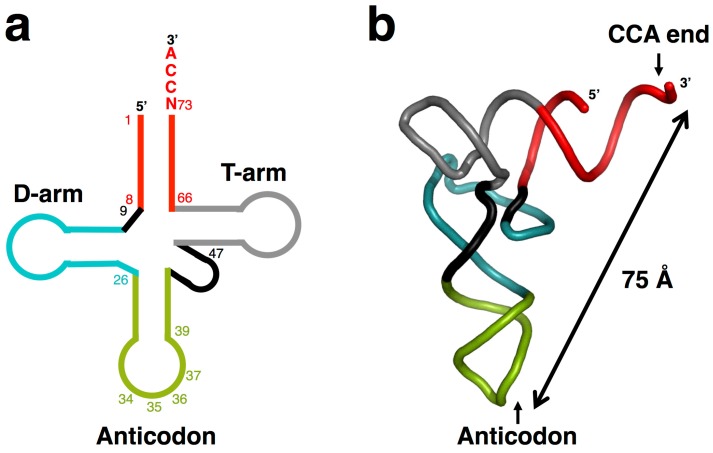
Secondary (**a**) and tertiary (**b**) structures of transfer RNA (tRNA). The cloverleaf secondary structure folds to the L-shaped tertiary structure; both structures have corresponding colors. tRNA has the universal single-stranded CCA-3′ terminus and a neighboring nucleotide at position 73 called “discriminator”, which is also non-base-paired in most cases. Several positions are numbered to facilitate the discussion.

Francis Crick once remarked that transfer RNA (tRNA) looks like nature’s attempt to make RNA do the job of a protein [1]. tRNA, discovered by Paul Zamecnik and collaborators [2], is a literal “adaptor” molecule [3] that mediates the translation of information from messenger RNAs (mRNAs). tRNA was the first non-coding RNA to be discovered. Now, our knowledge of the functions of non-coding RNAs has expanded drastically, especially in the area of microRNAs [4]. Modern tRNA is typically composed of ~76 nucleotides with the universal CCA-3′ terminus [5]. The cloverleaf secondary structure of a tRNA molecule folds into the L-shaped three-dimensional conformation through complex tertiary interactions, including those between the D-arm and T-arm (Figure 1). Each arm of the L-shaped tRNA structure is composed of the acceptor stem plus T-stem/loop, and the D-stem/loop plus anticodon stem/loop, respectively [6,7]. An amino acid is attached at the 3′-end of the acceptor stem and a trinucleotide anticodon is located in the anticodon loop. The distance between the ends of the two arms is about 75 Å (Figure 1).

The classic “chicken-or-egg” conundrum in molecular biology seemed to be solved by the RNA world hypothesis [8], which was postulated following the discovery of ribozymes [9,10]. The RNA world is believed to account for the development of primitive life on Earth. However, could tRNA have also existed in the primitive forms of life?

## 2. Emergence of a Self-Replicating Unit

**Figure 2:**
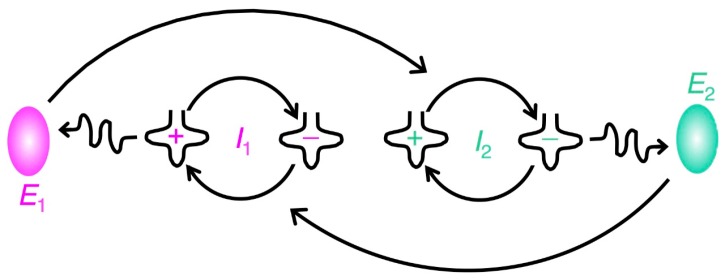
A simple model of a hypercycle. An RNA molecule (information carriers *I*_1_) not only instructs its own reproduction but also directs the formation of an intermediate *E*_1_, which contributes the catalytic aid for the reproduction of another RNA molecule (information carrier *I*_2_). *I*_2_ also has catalytic ability for both self-production and *E*_2_ formation, which facilitates *I*_1_ reproduction. These processes strengthen the resistance to mutations.

The ability to self-replicate is a crucial characteristic for defining life itself. Without coupled self-replicators, only a limited amount of information can be passed on from generation to generation. Therefore, when such self-replicating molecules emerged, what was their maximum size? As the size of these molecules increases, their complexity and stored information also increase. The point is whether such primitive self-replicating molecules can self-replicate within the range of an information error threshold that is intrinsically contingent on the molecules in the system. Manfred Eigen estimated the error threshold from the physical property inherent to nucleotides, which constitute unmodified RNA (*i.e.*, A, U, G, or C). He studied the relation between the replication fidelity and genome sizes, and defined the breakdown of correct replication as “error catastrophe” [11]. In order to increase the length of nucleotide chains while maintaining accurate replication, a set of cooperating replicators needs to appear. The “hypercycle” is composed of RNAs and enzymes that work cooperatively in a self-reproducing system (Figure 2). Here, the *i*-th RNA codes for the *i*-th enzyme *E_i_* (*i* = 1, 2, ..., *n*) and *E_i_* increases the replication rate of *I_i_*_+1_ in a cyclical manner, which means that *E_n_* eventually increases the replication rate of *I*_1_. (It does not matter whether *E_i_* is a ribozyme or a protein enzyme as long as it plays the cooperative role, although Eigen may have envisaged a protein enzyme.) The cyclic behavior of the hypercycle enhances the stability of the system and enzymes increase the replication accuracy. Before the establishment of such hypercycle self-replicating system (*i.e.*, without the cooperating system), the length of nucleotide chains that could be accurately replicated under Darwinian selection was no more than about 100 nucleotides [12]. Eigen defined the single-digit quality factors which determine the accuracy of complementary instruction. Purine → purine and pyrimidine → pyrimidine substitutions are by far more frequent than any cross-type substitutions based on the experiments with RNA-replicases. Therefore, he assumed only one incorrect alternative for any position of the sequence and finally concluded that the properties inherent to A, U, G, or C effect a discrimination of complementary from non-complementary nucleotides with a quality factor not exceeding a value of 0.90 to 0.99 [12]. Although the length of the chain depends on the accuracy of the primordial replication, by using the threshold value estimated under these criteria, the “error catastrophe” occurs beyond more than around 100 nucleotides. Notably, this size is comparable to that of the tRNA molecule [12].

## 3. Features of tRNA Aminoacylation and Evolutionary Significance

The top half of the L-shaped tRNA structure containing the aminoacylation site is often called a “minihelix” (a coaxial stack of the acceptor stem on the T-stem of tRNA) [13,14], and the isolated domain can function as a substrate for aminoacylation by many aminoacyl tRNA synthetases (aaRSs) [15,16,17]. Current tRNA aminoacylation by an aaRS generally occurs via the following two consecutive reactions:

aa + ATP + aaRS → [aa−AMP]aaRS + PPi
(1)

**Figure 3:**
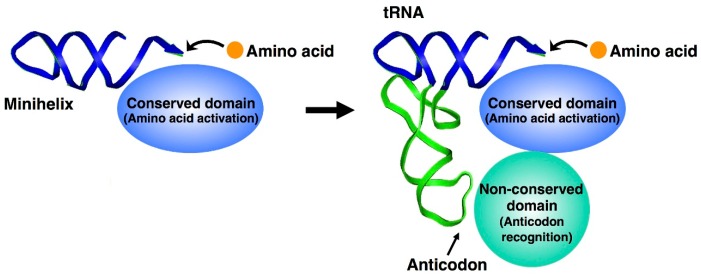
Evolution of aminoacyl tRNA synthetases (aaRSs) and its relation to tRNAs. The minihelix region (half domain of tRNA with the amino acid attachment site) interacts with the conserved domain of aaRSs for amino acid activation. The other tRNA half interacts with the non-conserved domain of aaRSs for specific recognition of an anticodon, although there are some exceptions, including tRNA^Ser^ and tRNA^Ala^ [21].

[aa−AMP]aaRS + tRNA → aa−tRNA + AMP + aaRS
(2)

where aminoacyl adenylate is formed as an intermediate in the form of a complex with aaRS (denoted as [aa−AMP]aaRS), and then the activated aminoacyl group is transferred from adenylate to the 3′-end of tRNA [18]. aaRSs are classified into two groups based on the amino acid sequence and catalytic domain structure [19]. Within each group, the amino acid activation domains of aaRSs are structurally similar, whereas the anticodon-binding domains possess more diverse structures [20]. The minihelix region interacts with the conserved domains of aaRSs and the anticodon-containing half interacts with the non-conserved domains (Figure 3). These structural features suggest that the conserved domain of aaRSs and one half of the L-shaped tRNA structure, a minihelix with the universal CCA-3′, appeared first, and the anticodon recognition domain of aaRSs and the other half of the L-shaped tRNA structure with anticodon were added later [13].

Class I synthetases possess active sites with the Rossmann fold motif and consensus sequences such as HIGH and KMSKS. In contrast, class II synthetases have active sites composed of antiparallel β-sheets with three motifs [22]. Although these classes may have two distinct evolutionary origins, the tertiary structures of aaRSs revealed that a member of each of the two classes can be docked simultaneously onto the opposite sides of the tRNA acceptor stem, thus suggesting a possible organization of the genetic code [23,24]. The amino acid-activating domains of Class I and II aaRSs may have been coded by opposite strands of the same gene [25,26,27]. However, the idea is somewhat based on the pre-existence of a coding system. In addition, the proposed ancestors of aaRSs (urzymes) are composed of still quite a large number of amino acids (~46) [27]. Therefore, non-coded protein synthesis based on the ligation of short peptides could have existed earlier.

## 4. Chiral-Selective Primordial tRNA Aminoacylation

**Figure 4:**
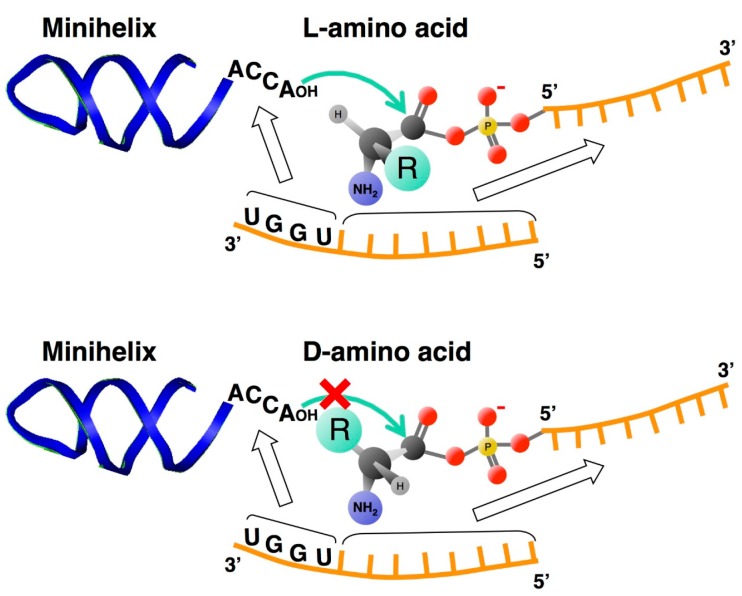
Chiral-selective aminoacylation of an RNA minihelix. Non-enzymatic aminoacylation with an aminoacyl phosphate oligonucleotide and a bridging nucleotide occurs with the clear preference for l- over d-amino acids. The arrow indicates nucleophilic attack of 3′-O of the terminal adenosine on the carbonyl carbon of the aminoacyl phosphate linkage. Chiral selectivity is likely due to steric hindrance of the side chain of d-amino acids.

The free energy change in aminoacyl adenylate hydrolysis is greater than that in aminoacyl tRNA (ester) hydrolysis [28], which means that the activated aminoacyl group can be spontaneously transferred from adenylate to the 3′-end of the tRNA (the second step being catalyzed by aaRSs in the modern biological systems). As the formation of aminoacyl adenylate has been proven to occur prebiotically [29], a model system of tRNA aminoacylation based on activated amino acids has been proposed [30]. In this system, the aminoacyl phosphate oligonucleotide and the universal CCA sequence at the 3′-end of the minihelix are in close proximity to each other and are bridged by another oligonucleotide. Surprisingly, the formation of an l-aminoacyl-minihelix was detected with a significant preference over that of a d-aminoacyl-minihelix, in the ratio of approximately 4:1 [30] (Figure 4). Chiral selectivity is likely due to the steric hindrance. The approach of oxygen (Nu) to the carbonyl carbon is described by the Bürgi-Dunitz angle defined as the Nu−C=O angle of approximately 105° [31] and the position of an amino acid side chain is crucial in determining chiral selectivity [32,33,34,35]. Here, it is clear that d-ribose RNA determines homochirality of l-amino acids, so the next question is about the origin of d-ribose in RNA. Template-directed auto-oligomerization using all possible combinations of homochiral and heterochiral diastereomers of short pyranosyl-RNA oligonucleotide 2′,3′-cyclophosphates occurred chiral-selectively, resulting in all d- or all l-products [36]. Therefore, similarly-produced RNAs could have been composed of all d- or all l-libraries. The important point here is that the sequences contained in the d- or l-libraries would not be the same, because the number of possible sequences would exceed the number of sequences actually formed during the process of extending RNA length. A specific sequence in only d-libraries may have exhibited an important unknown chemical propensity for establishing the RNA world; alternatively, it might have been chance that led to the use of l-amino acids in the translational system.

## 5. The CCA Sequence of tRNAs and the Origin of the Genetic Code

All tRNA molecules possess a single-stranded CCA sequence (C74C75A76) at their 3′-ends [5]. Weiner and Maizels proposed the genomic tag model, which postulates that tRNA-like structural motifs with CCA first evolved as 3′-terminal tags in RNA genomes for replication in the RNA world [37]. This model, therefore, hypothesizes that ancient linear RNA genomes also possessed tRNA-like structures with the 3′-terminal CCA as a replication initiation site [37]. The tRNA-like structures may have arisen early and played an essential role in the earliest replicating systems. Conserved throughout evolution, these structures are important in the contemporary world as well, as evidenced by their function in a wide variety of replicative processes in the modern biological systems [38,39,40].

Replication starting with GG could have been favored because of strong hydrogen bonds between G:C pairs (compared to A:U pairs) and strong stacking of the GG dinucleotide. Moreover, the addition of a non-templated 3′-terminal nucleotide (typically A), which commonly occurs in modern polymerases, would be favorable as the stacking of A stabilizes the terminal base pair [41,42,43].

**Figure 5:**
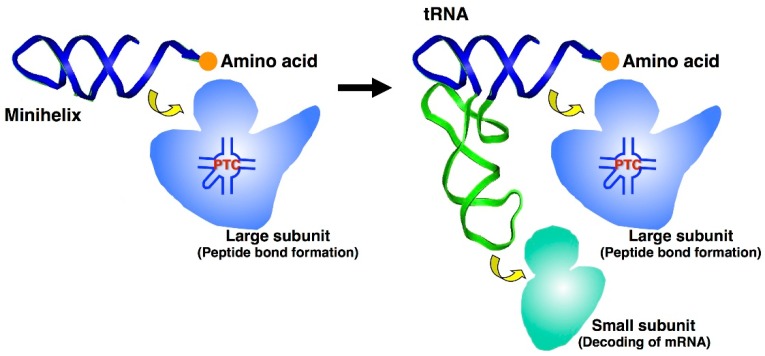
Evolution of ribosomes and its relation to tRNAs. The CCA end of the minihelix interacts with the large ribosomal subunit for peptide bond formation, and the end of the other tRNA half interacts with the small ribosomal subunit for decoding mRNA triplets through codon-anticodon interactions.

Similar to aaRSs, ribosomal subunits (large and small) are also functionally separated [44,45]. The CCA end of the minihelix domain of tRNA interacts with the large subunit, and peptide bond formation occurs at the peptidyl transferase center (PTC). In contrast, the end of the other tRNA half interacts with the small subunit and serves to decode mRNA triplets by codon-anticodon binding (Figure 5). The CCA sequence of tRNA is known to be important in the reaction with the ribosome [46]; in particular, the specific base-pairing of C74 and C75 with G2252 and G2251 in 23S rRNA (*Escherichia coli* numbering), respectively, are essential for peptidyl transferase activity [47]. Furthermore, A76 of tRNA^Val^ has been shown necessary for the editing activity of ValRS [48]. 

**Figure 6:**
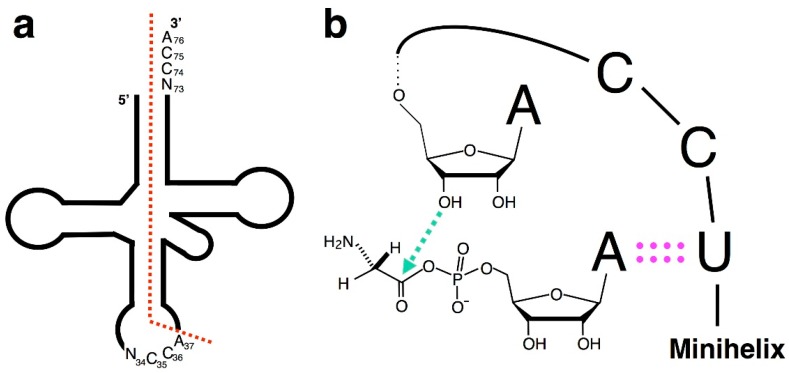
Plausible Gly assignment in the primordial genetic code. (**a**) The sequence of the full-length tRNA^Gly^ may be the product of tandem ligation of tRNA half with the NCCA-3′ terminus. The joint position corresponds exactly to the canonical intron insertion point; and (**b**) the UCCA-3′ terminus of the minihelix (or tRNA) forms a folded-back structure. The A of glycyl adenylate would base pair with the U in UCCA-3′, and nucleophilic attack of 3′-O of the terminal adenosine on the carbonyl carbon of the acyl phosphate group in glycyl adenylate would accomplish glycylation [52].

Recently, it has been proposed that an RNA with the CCA-3′ terminus was the origin of tRNA^Gly^ [49,50]. Considering the fact that the second and third nucleotides of tRNA^Gly^ anticodon are C35 and C36, respectively, and the following nucleotide is mostly adenosine (A37), half-sized hairpin-like RNA molecules with the CCA terminus might have been ligated to form full-length tRNA^Gly^ [49,50] (Figure 6a). A minihelix with the UCCA-3′ terminus is known to form a structure in which the 3′-sequence folds back [51] (Figure 6b). This is in contrast to a minihelix with the ACCA-3′ terminus, which does not adopt this conformation [43,51]. Glycyl adenylate could have been produced prebiotically, and the A would be expected to pair with the U in the UCCA-3′ of the minihelix [52]. The Gly residue would be transferred to the OH group of the 3′-terminal adenosine of the minihelix because of their close proximity in the folded-back structure (Figure 6b). The transfer is thermodynamically favorable.

There is a bias in base distribution at the discriminator base position (position 73), and more than half tRNAs have A at this position; U preceding CCA-3′ is not more common. However, eubacterial tRNA^Gly^ has the UCCA-3′ terminus (with U73 at the discriminator position), which may indicate a vestige of the primordial tRNA. Although the reaction can occur in all amino acids theoretically, plausible processes of the genetic code formation could be just briefly speculated below, based on the features of tRNA^Gly^ and the current genetic code.

First, in terms of prebiotic formation of aminoacyl adenylate, Gly is the most probable because it is not only the simplest and most abundant amino acid, but also has been shown to be formed in the conditions of the primitive Earth or even in space [53,54]. Second, in terms of the tRNA sequences, tRNA^Cys^ from all three kingdoms and tRNA^Gln^ from the eukaryotic cytoplasm also possess U73 [5]. However, Gln and Cys are produced from Glu and Ser, respectively, in biosynthetic pathways and both Gln and Cys could have been incorporated later in the coevolution theory of the genetic code [55,56]. Consistent with this idea, enzymes of aerobic thermophiles contain fewer or even no Cys residues [57,58,59,60]. In fact, the C_β_-S_γ_ bond of Cys is easily broken in aerobic and high temperature conditions [61]. Furthermore, the possibility that Lys or Arg were introduced first is not ruled out because they may have added chemical functionality to ribozymes, which required cations. However, Lys and Arg are biosynthesized from Asp and Glu, respectively, so they could have been incorporated later in evolution, similar to Gln and Cys [55,56].

The tRNA mutant with the GGA-3′ terminus (together with the C2252/C2251 double mutant in 23S rRNA) can function in peptidyl transfer [62]. Here, strong hydrogen bonding between G:C pairs would be retained in the interaction with the ribosome, but the tRNA with GGA-3′would not form the folded-back structure anymore. Hence, this mutant could not have been utilized in glycylation using glycyl adenylate.

So far, the detection of stereochemical interactions between amino acids and their coding nucleotides has not been fully successful and the results are inconclusive [63,64,65,66,67,68]. Therefore, the genetic code may have evolved from the assignment of Gly in the process beyond a strict frozen accident [52].

## 6. Origins of the Ribosome PTC and tRNA

The PTC is composed of only RNA molecules with the closest proteins detected approximately 18 Å away [47,69], which clearly suggests that the ribosome is a ribozyme [70]. The CCA termini of two tRNA molecules specifically interact with domain V of 23S rRNA, assembling the central part of the PTC, where the nucleophilic attack of the α-amino group of aminoacyl-tRNA on the carbonyl carbon of peptidyl-tRNA leads to the formation of a peptide bond [47]. A simple model system has also proved the occurrence of this reaction and validated its chemistry [71].

**Figure 7:**
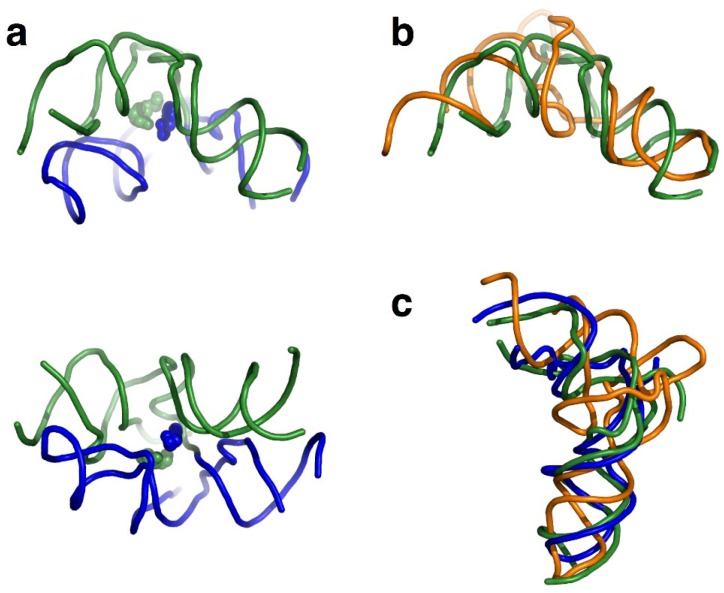
A putative mechanism of symmetrical primordial PTC formation. (**a**) The central loop of domain V in 23S rRNA forms a symmetrical pocket. The core unit structures from the *Haloarcula marismortui* 50S subunit (PDB code: 1VQN) are shown at two different angles, complexed with substrates mimicking the tip of tRNA 3′-end. The A- and P-core units are shown as blue and green spheres, respectively [73]; (**b**) the P-core unit overlaps well with tRNA (yellow; PDB code: 1EHZ); and (**c**) comparison of the shapes of the P-core unit, A-core unit, and tRNA after appropriate rotation.

Crystal structures of both bacterial and archaeal large ribosomal subunits clearly show that two L-shaped RNA units similar in size to tRNA form a symmetrical pocket [72,73] (Figure 7). The structure of the primordial PTC is likely associated with the formation of the peptide bond. Two L-shaped RNAs placed symmetrically could have functioned as a scaffold for proper positioning of the two reactant RNA molecules [44,72,73] (Figure 7). Duplication of half-sized hairpin-like RNA molecules might have produced some tRNA-like structures and, ultimately, could have formed the PTC. In addition, other tRNA-like molecules could have evolved to function as “adaptors.” The proto-PTC may have assisted symmetrical association of the two CCA units, favoring peptide bond formation [44,72,73].

In the modern translational system, the distance between the CCA terminus and tRNA anticodon (~75 Å) (Figure 1) is quite important to ensure the perfect alignment of both structures within the ribosome. The L-shape of tRNA is also required for the interaction with protein factors at each step of the ribosomal cycle [74]. Therefore, the L-shape of modern tRNA must be retained. Then, in the transition of the proto-ribosomes to modern ribosomes, proteins must have taken the functional place of RNA, as evidenced by release factors that mimic tRNA structure [75,76].

## 7. Evolution of the Genetic Code and tRNA

Rodin and Ohno have suggested the process of short RNA extension to the modern tRNA-like cloverleaf [77]. The 11 base pair-long double-stranded palindrome with complementary triplets in the center, each flanked by NCCA-3′ and 5′-UGGN, can replicate into a similar 25 base pair-long palindrome with a complementary triplet in the middle, after the addition of the folded-back triplet hook. Then, self-templating elongation of both palindromes should result in the formation of two helices with internal complementary triplets. Finally, the removal of 5′-UGGN from the 5′-terminus produces the modern tRNA-like cloverleaf. In this process, an intermediate hairpin places in the center a single-stranded triplet (plausible anticodon), which might represent the original anticodon-codon pair at the 1-2-3 positions of current tRNAs [77].

tRNA^Ala^ has a unique G3U70 base pair in the acceptor stem, and the wobble base pair has been shown to be a critical recognition site by AlaRS [78,79]. If the modern tRNA-aaRS system has evolved from the primordial tRNA-aaRS system, the primordial genetic code should have resided in the minihelix region and the recognition domain should have been also located in the primitive aaRSs, before the establishment of the universal genetic code. From this standpoint, G3U70 may be a vestige of the primordial genetic code, which may be called “operational RNA code” [13,14] and is thought to precede the universal genetic code referred to by de Duve as “the second genetic code” [80].

**Figure 8:**
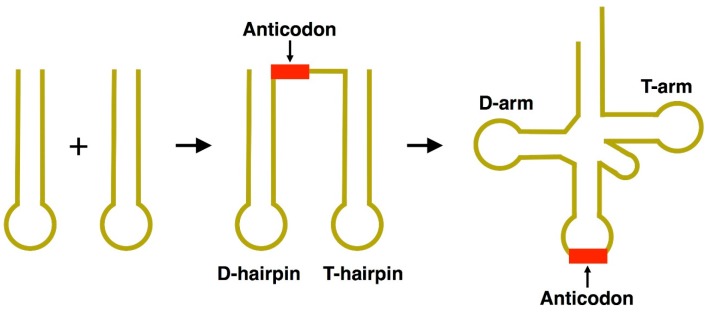
The double-hairpin model of tRNA formation. The acceptor stem bases and anticodon stem/loop bases in tRNA 5′-half and 3′-half fit the double-hairpin folding, suggesting that the primordial double-hairpin RNA molecules could have evolved to the structure of modern tRNA [81,82]. The anticodon is adjacent to the “D-hairpin” [82].

The number of possible RNA sequences composed of four nucleotide bases is 4*^n^* (where *n* is the number of nucleotides). In terms of genuinely mathematical estimation without any consideration of functional frequency, the total mass needed for the formation of nucleotide chain of the length similar to that of modern tRNA (~76 nucleotides) would be around ¹⁄₂₅th of the total mass of the Earth. However, for half-tRNA (~35 nucleotides), the required mass would be at most 100 g, suggesting plausibility of the formation of modern tRNAs via ligation of two half-sized hairpin-like RNA molecules (Figure 8). Several studies on the origin of the tRNA molecule, including statistical analysis, also indicate that most tRNA sequences have vestiges of double-hairpin duplication [81,82,83,84]. Pairing between the acceptor stem bases and anticodon stem/loop bases in the 5′-half and the 3′-half molecules of tRNAs fit the double hairpin folding. Helices can be formed by the acceptor stem region (mainly N1–N8) and the anticodon stem/loop region (N26–N34), and by the acceptor stem region (N66–N73) and the anticodon stem/loop with extra loop region (N39–N47); they should be located near or on the retained D- or T-stem/loop [82]. This fact strongly suggests that the double hairpin formation in the ancient prebiotic world underlies the evolution to the modern tRNA structure (Figure 1 and Figure 8).

Thus, the contemporary full-length tRNA molecules could have been formed by the ligation of half-sized hairpin-like RNA structures (Figure 8). In the evolution of codon assignments, it is important to minimize the effects of mutations, *i.e.*, similar amino acids are assigned in close positions [85]. If half-sized tRNA with the UCCA-3′ terminus is the ancestral molecules of tRNA^Gly^ (Figure 6a), Gly might be a strong candidate for the first piece of the genetic code [52,86] and tRNA^Gly^ might represent a vestige of the primordial tRNA. In this picture, Gly-containing primitive peptides could have preceded the protein-based evolution of life. β-turns stabilized by β-sheets are thought to be plausible active sites of early enzymes [87], and Gly together with Pro are often present in β-turns [68,88,89]. Pro has also been shown to destruct the G-quartet structure, and NGG (the anticodon of tRNA^Pro^) may have been involved in the establishment of the genetic code through Pro-dependent G-quartet formation/destruction [68]. Interestingly, G-quadruplex motifs are found at both the 5′-termini of 5′-untranslated regions and immediately after the 3′-termini of coding sequences, suggesting their important role in translation as well as transcription [90]. In addition, G-quadruplex has been suggested to be a primordial scaffold for peptide bond formation using aminoacyl-tRNAs [91].

Hydrophobic binding energy of a methylene group is about 1 kcal/mol, and the probability of an amino acid to be replaced by another one with additional methylene group would be about ⅕ [92]. Although modern aaRSs achieve strict discrimination between these two different amino acids using editing mechanisms (error rate as low as ¹⁄₄₀,₀₀₀ [93]), a simple thermodynamic process alone makes it impossible to discriminate with such a high fidelity. Therefore, the primordial genetic code could have been non-selective for several sets of amino acids with similar side chains. Gly (and also Ala, a similar amino acid with an additional methylene group) and Pro might have been the first candidates assigned by the primordial genetic code [52,68]. Strict discrimination could have evolved with the development of a biosynthetic system for amino acids and an editing mechanism involving aaRSs.

## 8. Concluding Remarks

After the discovery of tRNA by Zamecnik, Crick refused to believe that it was indeed the adaptor since the size was much bigger than he had expected. In fact, tRNA could have originated from a smaller-sized RNA. However, the question remains as to whether the minihelix is the real progenitor of modern tRNA. Probably, the most primitive tRNA was composed of oligonucleotides similar in size to that proposed by Rodin and Ohno [77]. What is the real origin of the genetic code? What happened during the evolution of the full-length tRNA from a primitive tRNA? These are still critical issues that should be further investigated.
